# Effect of Fluid Thickening with a Gum-Based Thickening Product in Older Patients with Structural or Mild Oropharyngeal Dysphagia

**DOI:** 10.3390/nu18071138

**Published:** 2026-04-01

**Authors:** Johana Muchová, Mireia Bolívar-Prados, Adrián Núñez-Lara, Noemí Tomsen, Pere Clavé

**Affiliations:** 1 Gastrointestinal Physiology Laboratory, Hospital Universitari de Mataró, Universitat Autònoma de Barcelona, 08304 Mataró, Spainntomsen@csdm.cat (N.T.); 2 Centro de Investigación Biomédica en Red de Enfermedades Hepáticas y Digestivas (Ciberehd), 28029 Madrid, Spain

**Keywords:** structural dysphagia, mild dysphagia, thickening product, swallowing safety, swallowing efficacy, shear viscosity

## Abstract

**Background:** The effect of fluid thickening in older patients with oropharyngeal dysphagia (OD) is not settled in the case of mild OD or OD caused by structural abnormalities. **Objective:** To assess the therapeutic effect and mechanism of action of the xanthan-gum-based thickener Tsururinko Quickly in older patients with structural OD and those with mild OD (Penetration–Aspiration Score < 3). **Patients and Methods:** We included 25 participants in each group (81.8 ± 7.1 vs. 77.4 ± 7.2 yr, respectively). Participants underwent videofluoroscopy (VFS) while swallowing 10 mL boluses at <50 mPa·s, 100, 200, 400, 800, and 1600 mPa·s to evaluate the safety and efficacy of swallowing and the biomechanics of the swallowing response at each viscosity level. After 30 s oral incubation, the effect of salivary α-amylase on shear viscosity was assessed using viscometer measurements. **Results:** (a) For the <50 mPa·s liquid series, no aspirations occurred in either group; however, 44% of patients with structural OD and 30% of patients with mild OD showed PAS 2 penetrations. (b) Fluid thickening reduced prevalence of penetrations with a maximal effect at 800 mPa·s and without affecting oral or pharyngeal residue in either group. (c) Increasing shear viscosity did not affect timing of airway protection mechanisms nor bolus kinematics. (d) Oral incubation decreased viscosity by 1.7–1.8% at 800 mPa·s. **Conclusions:** Fluid thickening with TQ enhances swallowing safety in older patients with structural causes of OD and those with mild OD through compensatory mechanisms and without a consistent increase in pharyngeal residue across the tested viscosity range.

## 1. Introduction

The natural shift in swallowing capabilities due to aging is known as presbyphagia. This condition stems from a combination of physiological, functional, anatomical, and psychological changes that reflect a natural decline of functional reserve and is often associated with general frailty in older adults [1]. While these progressive alterations may increase the likelihood of developing dysphagia, they do not inherently result in swallowing impairment in otherwise-healthy older adults. As the prevalence of diseases increases with advancing age, the prevalence of oropharyngeal dysphagia (OD) likewise increases. Loss of muscle mass and strength, decreased tissue elasticity, diminished salivary secretion, reduced oral and pharyngeal sensitivity, declines in olfactory and gustatory function, decreased neural compensatory capacity of the aging brain, and impaired dental status, and osteomuscular changes in the cervical spine all increase susceptibility to OD and may precipitate the transition from presbyphagia to mild OD and further progression to severe OD [2]. The Dysphagia Working Group, a committee of members from the European Society for Swallowing Disorders and the European Union Geriatric Medicine Society, highlighted in a position statement that OD can be considered a geriatric syndrome. It is highly prevalent among older adults, results from multiple interacting factors, is associated with numerous comorbidities and poor prognosis, and needs a comprehensive, multidimensional treatment strategy [3]. OD is a very frequent condition in the elderly, affecting approximately 36.5% of patients in hospitals, 42.5% of patients in rehabilitation settings, and 50.2% of nursing home residents [4].

While OD is often a functional disorder associated with aging or systemic or neurological diseases (neurogenic dysphagia) [5,6], it can also arise from various structural alterations that obstruct bolus progression (peripheral “mechanical” dysphagia) [7]. The most common structural abnormalities include cricopharyngeal (CP) bar, cervical webs, osteophytes, Zenker’s diverticulum, skeletal abnormalities, and oropharyngeal tumors [8]. Anterior cervical osteophytes are frequently observed in the geriatric population, though they are rarely symptomatic [9]. CP is characterized by a posterior indentation of the esophageal lumen between cervical vertebrae 3 and 6 causing, which narrows the upper sphincter during deglutition [10].

Despite the possibility of surgical treatment and emerging neurorehabilitation options [11,12,13], the established approach to oropharyngeal dysphagia primarily relies on compensatory management. These measures include enhancing fluid viscosity by adding thickening products (TPs) [14,15,16,17,18], and employing postures and maneuvers [19,20].

TPs are commonly used to modify the diet of patients with OD by increasing the shear viscosity of the fluids they are mixed with. Several clinical trials (CTs) have demonstrated that over 90% of the population suffering from OD can be safely treated with just two viscosity levels: 200–250 mPa·s and 800–1000 mPa·s [17,21,22]. However, TPs are often prescribed and commercialized using qualitative viscosity descriptors such as nectar, honey and pudding or numeric levels (IDDSI) without SI units of measurement or robust scientific evidence [22]. In addition, the most common phenotype of OD patients included in CTs are older, post-stroke or those with neurodegenerative diseases such as Parkinson’s. Patients with mild and structural dysphagia are typically excluded from CTs. Therefore, further research with larger samples and a broader range of patient phenotypes with dysphagia is needed to obtain robust results and mitigate risks associated with complications of dysphagia.

The various types of TPs differ in their basic thickening agents (starches, gums, proteins and mixed products), but have the common aim of increasing the shear viscosity of alimentary fluids. Tsururinko Quickly (TQ) is a specific xanthan-gum-based formulation that incorporates trisodium citrate, calcium lactate, and dextrin. Our recent investigation into its rheological behavior and clinical impact—detailed in Shear-Viscosity-Dependent Effect of a Gum-Based Thickening Product on the Safety of Swallowing in Older Patients with Severe Oropharyngeal Dysphagia—demonstrated that TQ significantly bolsters swallowing safety within a 100–800 mPa·s range in older patients with severe OD [17]. This improvement follows a viscosity-dependent pattern and occurs without a relevant increase in pharyngeal residue. The physiological mechanisms behind TQ’s efficacy vary with viscosity: at low viscosity levels (100–200 mPa·s), the therapeutic effect of TQ is mediated by its intrinsic rheological properties; at viscosities of ≥ 400 mPa·s, improvements are observed in airway protection mechanisms and the time to laryngeal vestibule closure (LVC), and at viscosities ≥ 800 mPa·s, bolus transit velocity also slows down. Research further indicates that TQ remains stable against salivary amylase and maintains high patient tolerability [22]. Practically, employing a strategy focused on two primary viscosity targets of 200 and 800 mPa·s can ensure safe, effective swallowing for the majority of elderly patients with severe safety impairments [17]. In this previous study, “safe swallowing” was characterized by a Penetration–Aspiration Scale (PAS) score below 3. The PAS is a standardized scale used to quantify the extent of airway invasion during videofluoroscopic swallowing studies: (a) PAS 2 score refers to penetrations with material that enters the airway, remains above the vocal folds and finally is ejected from the airway, and (b) most studies use PAS scores of 1–2 to define safe swallows, while scores of 3–8 are used to define unsafe swallows [23]. Despite these definitions, clinicians increasingly question whether viscosity adjustment is necessary for seemingly “safe” swallows (PAS 1–2) or for structural dysphagia, so a focused analysis is warranted.

Taken together, previous studies have defined viscosity-dependent benefits primarily in moderate to severe neurologic OD, often overlooking milder and structural phenotypes [3,18,21,24] and structural cases have shown variable or even paradoxical responses in early studies [25]. This paper draws on the prospective, single-centre trial that demonstrated the viscosity-dependent efficacy of Tsururinko Quickly (TQ) in severe OD [17]. Whereas the primary report analysed patients with PAS ≥ 3, this exploratory secondary study examines two under-studied phenotypes—mild dysphagia (PAS 1–2) and dysphagia caused by structural abnormalities—whose optimal management remains uncertain. It presents the first prespecified viscosity–dose analysis in these groups, using a validated thickener/viscosity protocol derived from severe OD. Analysing the rigorously collected data from participants who were enrolled but not previously reported maximises scientific yield while sparing additional patients from radiation or other study procedures and allows us to determine whether TQ’s benefits extend to these groups.

Accordingly, our goal is to evaluate the therapeutic effect of the xanthan gum thickener Tsururinko Quickly (TQ, Morinaga Milk Co., Tokyo, Japan) at five viscosities (100, 200, 400, 800, and 1600 mPa·s) in older adults with structural abnormalities detected in the oropharynx and those with mild OD (PAS 1–2, with or without oropharyngeal residue), and to clarify both its mechanism of action and the influence of oral processing on its rheological properties in these populations.

## 2. Patients, Materials and Methods

### 2.1. Experimental Design and Study Population

**Figure 1:**
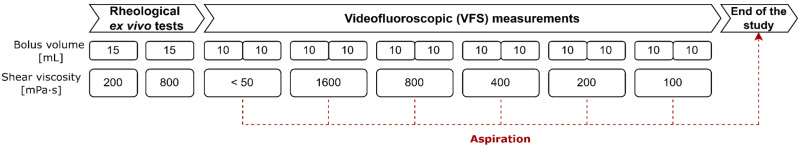
Schematic representation of the NCT04565587 trial design at Consorci Sanitari del Maresme.

This study was an interventional, non-randomized, multiple-dose, fixed-order, single-center investigation aimed at analyzing the therapeutic effect of a TP by videofluoroscopy (VFS) in patients exhibiting mild OD, and patients suffering from structural OD such as those with OD associated with osteophytes or CP bar. The main study providing data for this substudy was registered on the Clinical Trials Gov website under code: NCT04565587 (Date: 25 August 2020). The present analysis represents a post hoc exploratory subgroup analysis of these data, in which patients were stratified according to the presence of mild versus structural OD. The entire study procedure was conducted within a single visit (Figure 1).

Patients more than 70 years old with signs and symptoms of OD were enrolled in the study based on the following inclusion criteria:

1. For the group of patients with structural OD, inclusion criteria were OD associated with the presence of structural alterations, such as osteophytes, Zenker’s diverticulum, CP bars, and signing an informed consent to participate. Exclusion criteria included having OD symptoms not associated with structural abnormality, having dementia or severe cognitive disorders causing an inability to comply with protocol requirements, and allergy to any study product.
2. The inclusion criteria for the mild OD group were based on the baseline VFS assessment. Patients were classified as having mild OD if they presented a PAS score of 1 or 2 (1—material does not enter the airway; 2—material enters the airway, remains above the vocal folds, and is ejected from the airway) at all tested viscosities during this initial evaluation [26] and/or exhibiting signs of impaired swallowing efficacy [27]. Exclusion criteria included impaired safety of swallow in VFS study (PAS ≥ 3), dementia, severe cognitive disorders causing an inability to comply with protocol requirements, and allergy to any study ingredient.

The study protocol, participant information, and informed consent form was approved by the Ethics Committee of the CSdM under code 59/18. The study was conducted in accordance with the principles of the ‘World Medical Association Declaration of Helsinki’ (2013) and the International Conference on Harmonization (ICH) Guidelines for Good Clinical Practice (GCP, September 1997), as well as the Spanish regulations governing nutritional products applicable at the study site.

Patients were recruited between August 2020 and August 2022 at the Gastrointestinal Physiology Laboratory of Hospital de Mataró, Consorci Sanitari del Maresme (CSdM), Catalonia, Spain.

### 2.2. Products

The thickening product (TP) used for the study was Tsururinko Quickly (TQ), manufactured by Morinaga Milk Industry, Co., Ltd., Tokyo, Japan. TQ is composed primarily of xanthan gum, trisodium citrate, calcium lactate, and dextrin, with the following nutrient content per 100 g: 270 kcal, 0.5 g protein, 88.90 g carbohydrates, 21.90 g fiber, 960 mg sodium, 980 mg potassium, 30 mg phosphorus, 4.50 g ash, and 6.10 g water. Mineral water (Font D’Or, Vichy Catalan Corporation, Barcelona, Spain) was used for solution preparation. For VFS studies, the X-ray contrast agent Omnipaque™ (GE Healthcare Bio-Sciences, S.A.U., Madrid, Spain) was used. The swallowing of boluses at six shear viscosity levels (<50, 100, 200, 400, 800, and 1600 mPa·s) was examined through VFS following a standardized preparation protocol [22]. For VFS, the contrast solution was prepared by mixing 25 mL of Omnipaque™ with 25 mL of water and adding 0.00, 0.58, 1.00, 1.45, 2.45 and 4.30 g of TQ to obtain viscosities of <50, 100, 200, 400, 800, and 1600 mPa·s, respectively. For rheological testing, solutions were prepared using 100 mL of mineral water and the corresponding amounts of TQ. The TQ doses for each viscosity level were determined based on our previous study, which characterized their rheological properties [17].

### 2.3. Equipment

Videofluoroscopic data were recorded at a frequency of 25 frames per second utilizing a Panasonic AG DVX-100B imaging system (Matsushita Electric Industrial Co., Ltd., Osaka, Japan) and paired with a Toshiba Super XT-20 Image Intensifier (Toshiba Medical Systems Europe, Zoetermeer, The Netherlands). To evaluate the oropharyngeal swallowing response (OSR) and individual swallows, the Swallowing Observer software v.1 (Image & Physiology SL, Barcelona, Spain) was employed, following established protocols from our laboratory [22].

Rheological assessments were conducted using a rotational Haake Viscotester^®^ 550 (Thermo Fisher Scientific, Waltham, MA, USA). Shear viscosity was measured at a controlled temperature of 25 °C via the Thermo Scientific (Waltham, MA, USA) thermal management system. Depending on the thickness of the sample, different rotors were selected: the MV1 rotor was used for viscosity of 200 mPa·s, whereas the SV1 rotor was utilized for viscosity of 800 mPa·s. These measurement techniques and rheological standards align with our previously published methodology [22]. To investigate how oral processing influences the rheological behavior of the thickened products (TPs), we performed a comparative analysis of viscosity before and after oral incubation for every participant. This analysis was conducted at two representative viscosity levels, 200 and 800 mPa·s. Following previously described methods, subjects held a 15 mL bolus of each viscosity level in the oral cavity for 30 s before expectorating the sample for viscometer analysis (Figure 1).

### 2.4. Videofluoroscopy

Swallowing function was evaluated via lateral-view imaging, capturing the oral cavity, pharyngeal space, and the proximal portion of the esophagus [8,27]. Following the safety protocol illustrated in Figure 1, participants were administered duplicate 10 mL boluses for each viscosity level. Administration started with thin liquids, followed by the highest viscosity, and then proceeded in decreasing order of thickness. This fixed sequence was used to prioritize patient safety and reduce the risk of early termination due to severe aspiration, in line with published VFS recommendations [28] and prior studies on viscosity modification in dysphagia. The trial was discontinued immediately if aspiration occurred with any thickened sample. The VFS recordings were reviewed for visuoperceptual indicators of safety impairment, specifically aspirations, penetrations, and the mean PAS score. For each viscosity level, the worst swallowing outcome observed across the two swallows was used for analysis. Swallowing efficacy was also monitored by documenting the presence of pharyngeal or oral residue, which was graded using a three-point scale 0 = no residue, 1 = coating residue, 2 = pooling residue [29]. Additionally, the biomechanical response of the OSR was quantified by measuring the duration until laryngeal vestibule closure (LVC) and the opening of the upper esophageal sphincter (UESO). For these biomechanical parameters, mean values across the two swallows were calculated. Bolus flow dynamics were also calculated, including the average and peak pharyngeal velocity as well as the kinetic energy of the bolus prior to UESO entry [27].

To monitor for delayed complications or adverse reactions, all participants received a follow-up telephone call 48 h after the completion of the VFS session.

### 2.5. Hedonic Scale

Immediately following each swallow during the VFS procedure, participants’ acceptance of the thickened product was evaluated using a 5-point facial Likert hedonic scale. Scores were recorded on a scale from 0 to 5 to quantify the sensory appeal of each sample.

### 2.6. Adverse Events

Adverse event severity was graded according to the Common Terminology Criteria for Adverse Events (CTCAE; U.S. Department of Health and Human Services, National Institutes of Health v5.0; Gastrointestinal Disorders). To establish the potential link between the study product and any reported incidents, the World Health Organization (WHO) and the Uppsala Monitoring Centre (UMC) Standardized Causality Assessment System were employed (“The Use of the WHO-UMC System for Standardized Case Causality Assessment”).

### 2.7. Outcome Parameters

The primary endpoint was the proportion of participants exhibiting PAS score 1 (material does not enter the airway) at each viscosity level [26].

Secondary endpoints included: the swallowing efficacy evaluated based on the frequency and degree of oral and/or pharyngeal residue [29], the rheological impact of oral incubation (simulating salivary amylase interaction at 50 s^−1^) and the effect of pharyngeal shear thinning at 300 s^−1^ on viscosity levels of 200 and 800 mPa·s.

### 2.8. Statistics and Data Analysis

This secondary analysis used the sample originally planned for the parent study. The sample size calculation was performed per cohort and was based on detecting a clinically relevant change in the proportion of patients achieving safe swallowing at the highest viscosity level. Specifically, we assumed an alpha risk of 0.05 and a beta risk of 0.20 (80% power) in a two-tailed model to detect an increase in the proportion of participants with penetration–aspiration <50 mPa·s from 0.15 at baseline to 0.65 at 100 mPa·s. Under these assumptions, 24 subjects were required in each cohort, allowing us to compare outcomes across repeated measures of viscosity. Anticipating a 10% attrition rate, we inflated the target sample accordingly.

The quantitative variables, such as bolus kinematics and oral motor responses, were reported as means ± standard deviation (SD). Differences across these groups were evaluated using non-parametric ANOVA methods (Friedman and Kruskal–Wallis), followed by Dunn’s post hoc test for multiple comparisons. For qualitative variables, including the prevalence of swallowing safety and efficacy markers, data were expressed as absolute frequencies and percentages. Global differences were assessed using the chi-square test, while specific 1:1 comparisons were performed via Fisher’s exact test. A *p*-value of less than 0.05 was considered the threshold for statistical significance. All data processing and statistical evaluations were performed using GraphPad Prism 8.0 software.

## 3. Results

### 3.1. Demographics and Patient’s Clinical Characteristics

The present study is a substudy of a group of patients not included in our main CT NCT04565587 [17]. In that CT, a total of 305 patients were screened and participated in the VFS study. Among these, 85 participants (27.9%) accomplished the inclusion criteria of severe OD as they presented PAS ≥ 3 and were enrolled in the study [17]. A total of 170 patients were excluded for various reasons, including inability to complete the VFS protocol, failure to maintain the required seated position, nausea, or withdrawal due to equipment malfunctions and technical issues.

**Figure 2:**
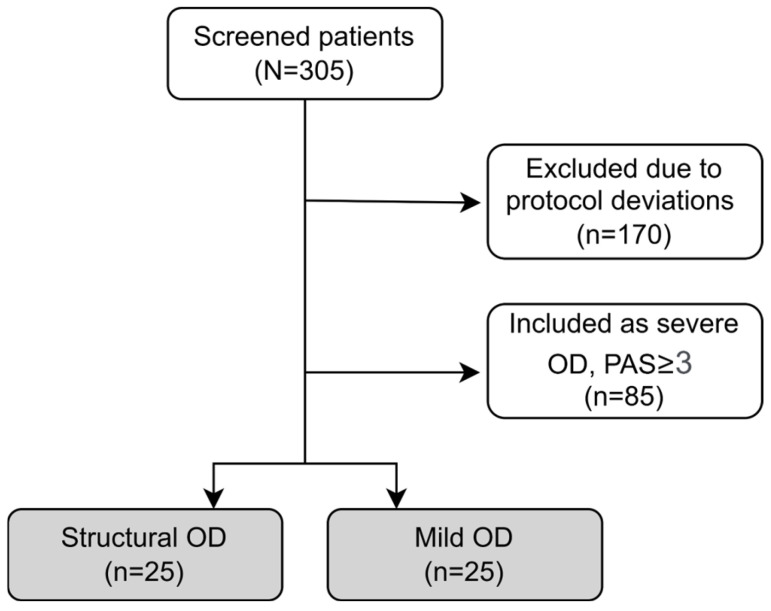
Flow diagram illustrating the process of subject recruitment and study enrollment. PAS indicates the penetration–aspiration scale; OD, oropharyngeal dysphagia.

The remaining 50 patients with swallowing impairments were divided into two groups according to OD etiology (Figure 2) and included in this study: (a), a group of 25 patients with OD from structural causes (mainly osteophytes or CP bars), (b) a group of 25 participants with mild OD, PAS scores 1 or 2 and exhibiting swallowing efficacy impairments.

The mean age of the study population for patients with structural OD was 82 ± 7 years, with a higher prevalence among women (72%). Among the causes of structural OD, 60% were associated with cervical osteophytes, 20% with CP bar, and 20% of patients had both osteophytes and CP bar (Table 1). The study population for participants characterized by mild OD consisted of 54% males, and the mean age was 77 ± 7 years, and these parameters were significantly different from the group with structural OD. Nutritional status evaluation using MNA-sf showed high percentages of patients who were malnourished or at risk of malnutrition in these two groups: 38.1% for the structural OD group and 63.2% for the mild OD group (Table 1).

No adverse events related to the study procedures or the investigational product were observed during the study. Furthermore, no delayed adverse events or complications were reported during the 48 h follow-up period.

### 3.2. Therapeutic Effect of Increasing Shear Viscosity

#### 3.2.1. Safety and Efficacy of Swallowing

The entire study population from both groups fell within PAS levels 1 and 2. Table 2 summarizes VFS signs across the studied populations, indicating that, although no aspirations occurred, penetrations were observed, and impaired efficacy was very prevalent.

**Figure 3:**
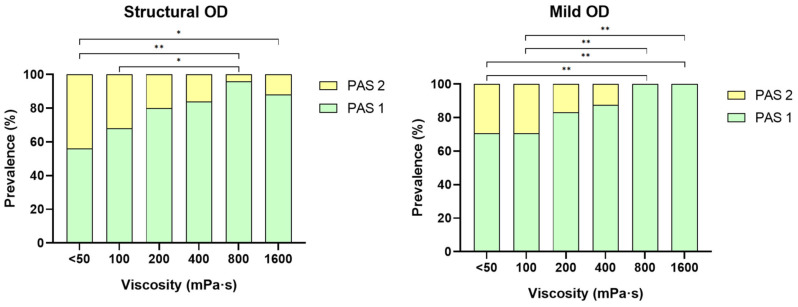
Prevalence of patients with structural OD (**left**) and mild OD (**right**) with swallows without entry of bolus into the larynx or trachea (PAS 1) at each viscosity level assessed. Significant improvements in safety were observed at 800 and 1600 mPa·s compared to thin liquids. * *p* < 0.05; ** *p* < 0.01. OD indicates oropharyngeal dysphagia; PAS, penetration–aspiration scale; PAS 2, bolus enters the airway, remains above the vocal folds, and finally is ejected from the airway.

VFS PAS signs of safety of swallow at each viscosity level are presented in Figure 3. Significant differences were observed between thin liquid (<50 mPa·s) and viscosity levels above 800 mPa·s for both population groups with structural or mild OD. Increasing the shear viscosity of the thickening product enhanced the prevalence of patients with a safe swallow in both groups. At 800 mPa·s, the maximal level of safety was achieved, with 96% of patients with swallows without entry of bolus into the larynx (PAS 1) for patients with structural OD, showing significant differences compared to thin liquids (*p* < 0.01). The same viscosity level achieved a maximal level of safety, reaching 100% in patients with mild dysphagia also significantly higher than that of thin fluids.

**Figure 4:**
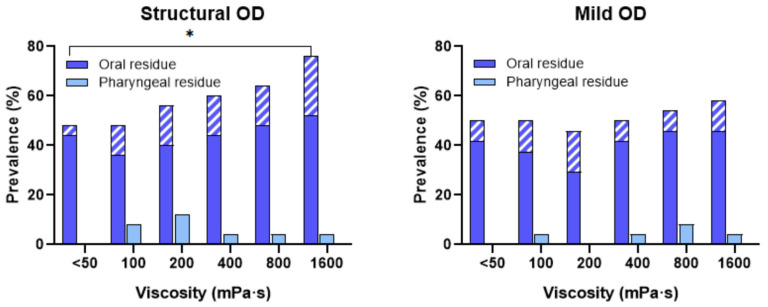
Prevalence of patients with structural OD (**left**) and mild OD (**right**) with oral, and pharyngeal residue at each viscosity level categorized by the Robins Scale (Coating vs. Pooling). The diagonal-lined segments of the bars represent pooling residue. OD indicates oropharyngeal dysphagia. * *p* < 0.05.

The prevalences of coating and pooling oral residues were 29–52% and 4–24%, respectively, with significant difference in patients with structural OD for oral pooling residues between viscosities < 50 and 1600 mPa·s. Oral residues were significantly more prevalent than those of pharyngeal residues. In contrast, no pooling pharyngeal residues were observed in any group and the prevalence of coating pharyngeal residues was 0–12% (Figure 4).

**Figure A1:**
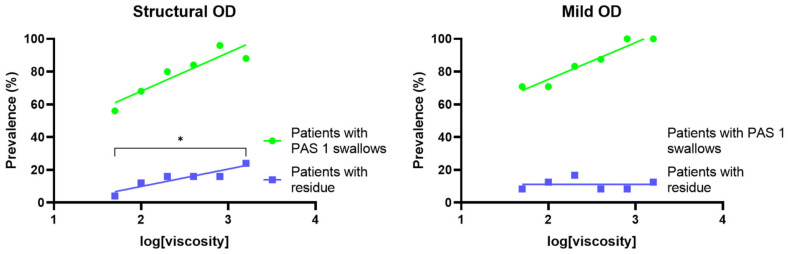
Dose–response curves. The effect of increasing viscosity on the prevalence of patients with PAS 1 swallow and swallows with pooled residue (oral and pharyngeal) in each phenotype of patients. OD indicates oropharyngeal dysphagia; PAS, penetration–aspiration scale; * *p* < 0.05.

The prevalence of patients with PAS 1 swallows, along with the prevalence of swallows with residue (oral or pharyngeal) including pooling, at each viscosity level is presented in Figure A1 in Appendix A. For patients with structural OD, there is higher risk of residue with increasing viscosity compared to those with mild OD.

#### 3.2.2. Oropharyngeal Swallowing Response

Patients from both populations included in this study exhibited impaired timing of OSR, with a delayed time to LVC (226.7 ± 115.3 ms for structural OD and 257.4 ± 112.5 ms for mild OD). These values are higher than those reported in older healthy volunteers (158 ± 41 ms for LVC) and remain below the delays described in patients with severe OD (363 ± 73 ms), according to Guanyabens et al. [30]. In contrast, time to UESO (165.9 ± 79.1 ms for structural OD and 189.5 ± 75.5 ms for mild OD) falls within the range of values reported in older healthy volunteers (206 ± 72 ms) in the same reference study.

**Figure 5:**
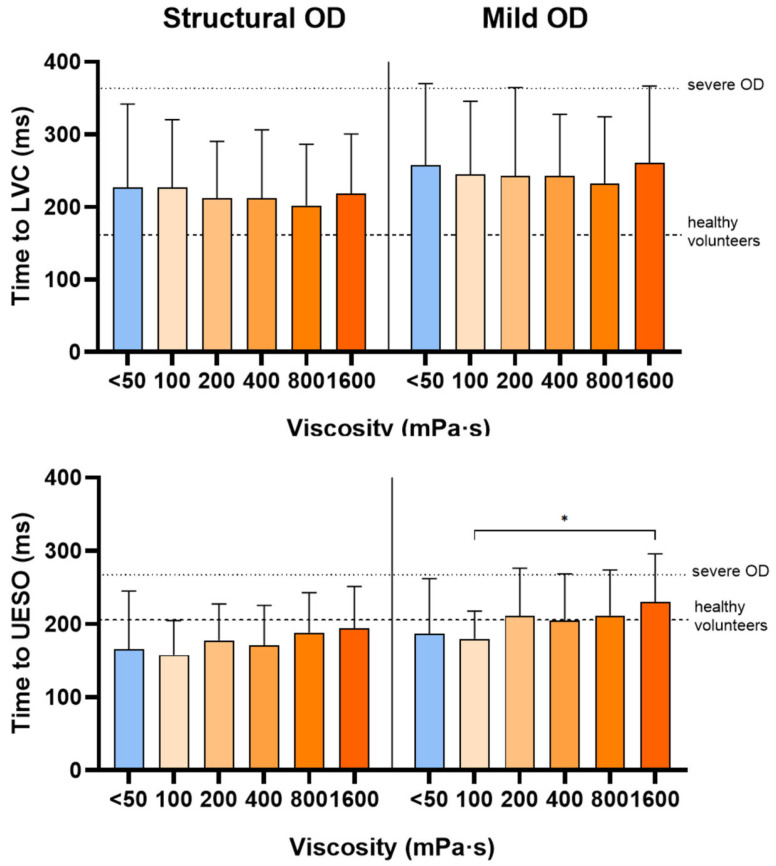
Influence of the bolus viscosity on timing of the oropharyngeal swallow response. The mean times to laryngeal vestibule closure (LVC) and upper esophageal sphincter opening (UESO) across the tested viscosity range are illustrated. * *p* < 0.05. Dashed lines represent the reference values reported in older healthy volunteers (158 ± 41 ms for LVC; 206 ± 72 ms for UESO), while dotted lines indicate reference values reported for older patients with severe OD (363 ± 73 ms for LVC; 267 ± 79 ms for UESO) according to Guanyabens et al. 2024 [30].

The timing of bolus transfer events for patients with structural OD was not affected by thickening the fluid with TQ. In patients with mild OD, a tendency toward delayed timing was observed. At the highest viscosity level (1600 mPa·s), a significant increase in the time to UESO (*p* < 0.05) was observed, as displayed in Figure 5.

Values for the kinematics of swallowing are presented in Table 3. The mean bolus velocity decreased with increasing viscosity, ranging from 0.41 ± 0.23 to 0.31 ± 0.19 m/s in structural OD and from 0.42 ± 0.28 to 0.27 ± 0.13 m/s in mild OD. A similar trend was observed for bolus kinetic energy, although these changes did not reach statistical significance.

#### 3.2.3. Hedonic Scale

**Figure 6:**
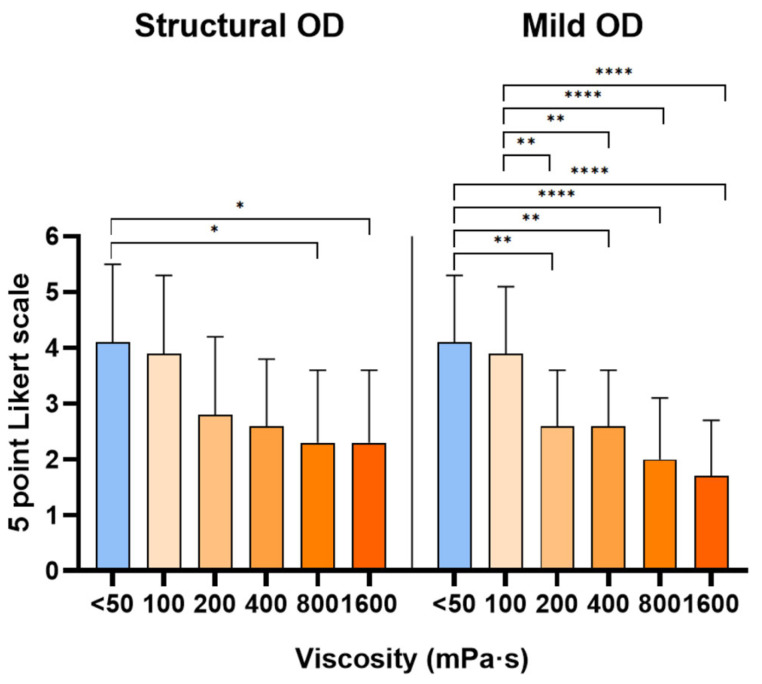
Mean ± standard deviation scores provided by participants using a 5-point facial Likert scale are displayed for each viscosity level assessed. * *p* < 0.05; ** *p* < 0.01; **** *p* < 0.0001.

The average ratings of palatability for each viscosity level evaluated in the structural and mild OD study populations is depicted in Figure 6.

The palatability of the samples significantly decreased as viscosity increased. There was no statistically significant difference between the two groups, as both groups preferred bolus viscosities of less than 50 and 100 mPa·s.

### 3.3. Rheological Characterization After Oral Incubation

In this study, bolus shear viscosity was measured for shear rates from 0 to 1000 s^−1^ for boluses pre- and post-oral incubation mimicking the effect of oral and pharyngeal phases of swallowing [22].

**Figure A2:**
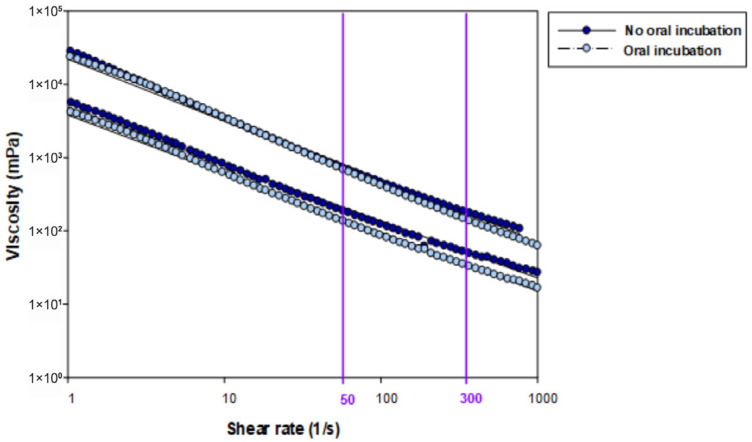
Viscosity flow curves of the pre- and post-oral incubation viscosity levels of 200 and 800 mPa·s, respectively, in a shear rate range from 1 to 1000 s^−1^.

After oral incubation and for the structural OD population, shear viscosity measurements at 50 s^−1^ were 155.6 ± 28.5 and 785.2 ± 63.0 mPa·s for target viscosity levels of 200 and 800 mPa·s, respectively. We observed a viscosity decrease of 22.2% for 200 mPa·s and 1.8% for 800 mPa·s, confirming amylase resistance of TQ. We observed similar results for the mild OD population post-oral incubation, with shear viscosity measurements at 50 s^−1^ of 150.0 ± 26.5 mPa·s with an amylase effect of 25.0% for target viscosity level of 200 mPa·s and 786.5 ± 52.5 mPa·s with an amylase effect of 1.7% for 800 mPa·s. Shear thinning at the pharyngeal shear rate led to a mean viscosity decrease of 75−79% at 300 s^−1^. Figure A2 in Appendix A shows the shear viscosity flow curves for samples with pre- and post-oral incubation viscosity levels of 200 and 800 mPa·s, respectively, over a shear rate range of 1 to 1000 s^−1^.

## 4. Discussion

In this study, we found that 44% of older patients with structural OD and 30% of older patients with mild OD attending our dysphagia clinic at Mataró Hospital, Catalonia, Spain, exhibited PAS 2 penetrations when swallowing thin liquids; PAS 2 penetrations are characterized by bolus material that enters the airway at the laryngeal vestibule, remains above the vocal folds and is ejected. These PAS 2 penetrations were associated in both groups with a delay in time to LVC, which is the main airway protection mechanism during swallowing. Additionally, both groups showed VFS signs of impaired swallowing efficacy with oral and/or pharyngeal residue. The study also explored the therapeutic effect of TQ on the biomechanics of the swallowing response in these two phenotypes of patients in the early stages of oropharyngeal dysphagia associated with aging. We found that fluid thickening with TQ enhanced swallowing safety in a viscosity-dependent manner in both groups. The thickened fluid with TQ achieved the highest safety at 800 mPa·s avoiding any entry of material into the laryngeal vestibule in 96–100% patients through compensatory mechanisms related to the intrinsic rheological properties of TQ, without relevant changes in swallow physiology and without any major impact on pharyngeal residue. This effect of TQ is observed within the therapeutic range of TQ (100–800 mPa·s) which was defined in our previous studies involving more severe older patients with OD [17], further supporting the strong therapeutic profile of this thickening agent.

Both the European Union Geriatric Medicine Society and the European Society for Swallowing Disorders have classified oropharyngeal dysphagia (OD) as a significant geriatric syndrome. This designation stems from its widespread occurrence in elderly populations and its strong association with severe health risks. These include lung infections related to aspiration, as well as nutritional and fluid deficits. Furthermore, untreated OD frequently leads to diminished physical function and increased frailty, often resulting in higher rates of hospital admission, long-term care placement, and overall unfavorable clinical outcomes [3]. At our institution, five studies involving 3328 patients were recently reviewed and analyzed. Our analysis identified OD as an independent and significant risk factor for multiple adverse outcomes. Specifically, geriatric patients diagnosed with OD faced mortality risks more than three times higher at one month (OR 3.28) and one year (OR 3.42) following a pneumonia episode. The data further revealed that the presence of OD increased the likelihood of malnutrition by 2.72 times and doubled the risk of lower respiratory tract infections (2.39-fold). OD was also linked to a 1.82-fold rise in general pneumonia readmissions and a five-fold (5.07) increase in readmissions specifically for aspiration pneumonia. These findings highlight the necessity for early detection, thorough clinical evaluation, and customized therapeutic interventions for these patients [31].

This exploratory subgroup analysis draws on the same prospective, single-centre trial that supported our previous report on severe oropharyngeal dysphagia [17]. Ethics approval, consent, videofluoroscopic swallow-study procedures, rheological testing of TQ (100–1 600 mPa·s) and safety monitoring were identical to the parent protocol. Data for two under-represented phenotypes—mild OD (PAS 1–2) and structural OD—were collected alongside the severe cohort using the same standard operating procedures. These groups were stratified post hoc to further investigate potential differences between phenotypes, as they frequently exhibit airway penetration or residue despite “safe” PAS scores and lack evidence-based viscosity targets. Most previous trials exclude these populations, leaving clinicians without guidance. Accordingly, we describe the therapeutic efficacy and underlying mechanistic insights of TQ in these mild and structural cases, complementing our earlier findings in severe OD. Together, the two publications provide a comprehensive viscosity–dose–response map across the full clinical spectrum of OD.

In the present sample, nutritional status evaluation using MNA-sf revealed high percentages of patients malnourished or at risk of malnutrition: 38.1% for the structural OD group and 63.2% for the mild OD group. Regarding safety, the high proportion of patients with PAS 2 penetrations, associated with a significant delay in time to LVC, indicates potential risk of aspiration and subsequent respiratory infections. This risk of aspiration pneumonia can dramatically increase when these aged patients suffer acute illnesses leading to decreased functional capacity and resilience, hospital admission, or progression of aging and age-associated neurodegenerative conditions accelerating the transition from presbyphagia to mild or severe OD [32]. In this setting, even mild signs of oropharyngeal dysphagia could lead to a deterioration in overall health status [33] and progress with aging.

The PAS categorization of safe swallowing during VFS studies has gained popularity in recent years, with researchers selecting different categorical boundaries along the 8-point scale and debating whether a PAS 2 represents “normal” or “pathological” airway invasion [34]. While we acknowledge that the categorization of PAS should be based on specific research design and questions, we suggest that these categorical PAS boundaries must also be interpreted within a physiologic framework. For example, transient PAS 2 penetrations can be observed in some healthy adults with normal swallow response and intact airway protection mechanisms, when they may be considered “normal” [34]. However, we believe this might not be the case in older patients with neurological or neurodegenerative conditions who present altered OSR and delayed airway protection mechanisms, signs of impaired efficacy of swallow, and/or structural causes of OD, as seen in both phenotypes in our study. We recently characterized the swallowing biomechanics and neurophysiology in older patients with OD and found that neither young nor older healthy adults presented any PAS 2 bolus penetrations into the laryngeal vestibule in contrast to 91% of older patients who were positive for OD in the V-VST clinical screening test, and presented PAS 2–5 penetrations and PAS 6–8 aspirations [30]. In addition, we found time to LVC in healthy older adults without OD was 158.4 ± 41.4 ms, which is significantly shorter than the 362.5 ± 73.3 ms in older patients with OD [30]. In the present study, the time to LVC was 226.7 ± 115.3 ms for patients with structural OD and 257.4 ± 112.5 ms for those with mild OD; and all of these patients also exhibited impaired efficacy and oropharyngeal residue, indicating they are not as “healthy” and are at certain risk for respiratory complications [24,35]. Our study addresses a relevant clinical question by showing that fluid thickening with TQ can reduce the occurrence of “pathological PAS 2 penetrations,” suggesting improved airway protection in patients with mild and structural OD.

The main results of the present study demonstrate that thickening fluids with TQ improve swallowing safety in a shear-viscosity-dependent manner by reducing the prevalence—and almost eliminating—these “pathological” PAS 2 bolus penetrations into the laryngeal vestibule for both populations—patients with structural and mild OD. This effect was achieved without a consistent increase in pharyngeal residue across the tested viscosity range, although oral residue remained frequent and some viscosity-dependent effects were observed.

In the present study, maximal therapeutic effect was observed at 800 a mPa·s, further increases in shear viscosity did not cause any increase in the therapeutic effect of TQ on safety of swallow. Similar results were achieved in our previous clinical study on older patients with more severe OD and aspirations [17]. Findings revealed that increasing bolus thickness with TQ led to a substantial and statistically significant enhancement in swallowing safety for elderly patients suffering from severe OD and aspirations. That study established a therapeutic range between 100 and 800 mPa·s. By analyzing the dose–response relationship of different thickness levels, we identified 800 mPa·s as the optimal shear viscosity for patient management; beyond this point, no additional clinical benefits in safety were observed. Furthermore, the data indicated that utilizing just two specific TQ viscosity levels—200 and 800 mPa·s—was sufficient to achieve safe swallowing outcomes for nearly all elderly individuals with severe dysphagia [17]. On the other hand, it is noteworthy that this substantial clinical improvement at 800 mPa·s in the present study occurred across just two PAS levels, indicating that the criteria used were more stringent to eliminate any entry of material into the larynx and therefore, the upper limit of the therapeutic range of TQ (800 mPa·s) is needed to reflect a significant difference within such a narrow PAS range.

Regarding swallowing efficacy in this study, increasing shear viscosity with TQ did not result in a consistent increase in pharyngeal residue compared with thin liquids. However, oral residue remained common, and at the highest viscosity level (1600 mPa·s), a significant increase in oral pooling was observed in patients with structural OD. This viscosity is above 800 mPa·s, the maximal therapeutic level of TQ. Our earlier research in patients with severe OD demonstrated that TQ was associated with a moderate increase in oral coating across most viscosity levels, while its impact on pharyngeal residue was minimal and lacked statistical significance [17]. Taken together, these findings suggest that TQ does not substantially increase pharyngeal residue within its optimal therapeutic range of 200–800 mPa·s, although oral residue may persist and increase at higher viscosities. Minimizing pharyngeal residue is critical, as its presence is a known risk factor for aspiration occurring after the swallow. This represents a limitation of traditional starch-based thickeners at high viscosities and highlights a potential advantage of xanthan gum-based products such as TQ [36,37].

Similar to our previous study, we explored the mechanisms potentially underlying the therapeutic effect of TQ. A short time to LVC is crucial for protecting the respiratory tract during swallowing [35]. Both groups of patients included in this study presented a moderate delay in LVC; in contrast, bolus propulsion forces, time to UESO and bolus velocity were still preserved when considered in the context of values reported for older healthy adults [30]. This suggests that at the early stages of OD, the neural impairments delaying LVC precede the muscular ones involved in bolus propulsion and pharyngeal clearance [30]. In the present study, fluid thickening with TQ did not induce significant changes in the timing of airway protection or bolus transfer events. These findings suggest that the therapeutic effect of TQ may be explained by its rheological properties, rather than by modifications of the swallowing response, which remains relatively preserved in these patients. This contrasts with our previous observations in patients with severe OD, in whom fluid thickening was associated with changes in LVC timing and bolus velocity [17,30].

From a mechanistic perspective, thickening agents such as TQ exhibit viscoelastic behavior, with distinct responses under shear and extensional flow conditions. While shear viscosity is known to improve swallowing safety—mainly by slowing bolus flow and reducing penetration–aspiration risk—extensional properties may also influence bolus transport through the pharynx and upper esophageal sphincter. In extensional flow, the bolus undergoes stretching and deformation, processes that require higher energy and are more sensitive to the internal structure of the material [38,39,40]. Overall, extensional flow is less discussed in the context of dysphagia, although it may play a crucial role in interpreting bolus swallowing [41,42]. Xanthan gum-based formulations, such as TQ, have been reported to exhibit higher extensional viscosity and longer relaxation times compared with other thickening agents, which may increase resistance to bolus elongation and flow [43]. In this context, the reduction in bolus velocity and the tendency toward increased residue observed at higher viscosities in both mild and structural OD could be interpreted as being consistent with increased resistance to extensional deformation. This effect may be particularly relevant in structural OD, where anatomical constraints already require higher propulsive forces, but may also contribute in mild OD, where patients may have limited ability to generate the additional forces required for bolus deformation.

In our previous work, we evaluated several rheological parameters of thickening products and their relationship with swallowing safety and efficacy in a large cohort of patients with OD. Shear viscosity was consistently associated with improved swallowing safety in a dose-dependent manner, although it also contributed to increased residue. In contrast, extensional deformation, adhesiveness, and cohesiveness were not significantly associated with clinical outcomes in that analysis [22]. Taken together, the present findings are consistent with a predominant role of shear viscosity and α-amylase resistance in the therapeutic effect of TQ, while suggesting that additional rheological properties, such as extensional behavior, may contribute to bolus transport dynamics under specific conditions.

Finally, the inverse relationship between bolus thickness and the sensory appeal of thickened products is a recognized challenge in dysphagia management. The findings of this study reinforce this, as participants noted reductions in palatability as the viscosity levels increased. It should be noted that the boluses used here were prepared for acute diagnostic testing (containing X-ray contrast media), which may not perfectly replicate the experience of consuming these products in a standard home or clinical setting. Nonetheless, patient preference and acceptance are critical factors when incorporating thickening agents into multimodal treatment plans for OD. Currently, pharmacological treatment is under development and entails sensory stimulation of TRP channels using TRP agonists, offering promise for patients with mild OD of non-structural origin [44,45]. On the other hand, for patients with structural OD, pharmacological treatment using TRP agonists would not be effective, leaving them with fewer options unless considering surgical treatment. Thickening fluids appear to be the only viable option for them, and to optimize compliance, clinicians should aim to prescribe the lowest effective viscosity, as determined by objective measures like VFS or bedside screening tools [18].

It is important to acknowledge that this study has some limitations. First, the relatively small sample size limits statistical power; larger studies would enable the use of methods appropriate for repeated measures (e.g., Cochran’s Q test or mixed-effects models) to better account for intra-individual correlations. In addition, evaluating other thickening products would enhance the generalizability of the findings. Moreover, the classification of patients within the “structural OD” group was based on the presence of anatomical alterations affecting bolus transit (e.g., osteophytes or cricopharyngeal bar), without quantitative criteria for anatomical level or severity, and concomitant non-structural causes were not formally assessed or excluded; given the likelihood of mixed etiologies in older patients, this may have influenced the results. Finally, assessing boluses using extensional rheometry could provide a more comprehensive characterization of rheological behavior by capturing the full flow profile of the tested products.

## 5. Conclusions

The therapeutic effect of TQ—a xanthan-gum-based thickener—and its mechanism of action as well as the effect of oral processing on its rheological properties were examined at increasing levels of viscosity in older patients with structural abnormalities and those with mild OD. Fluid thickening with TQ at 800 mPa·s was associated with the highest swallowing safety, with 96–100% of patients showing no material entry into the laryngeal vestibule, likely mediated by compensatory mechanisms. Importantly, this improvement in safety was achieved without a consistent increase in pharyngeal residue, although oral residue persisted in patients with structural OD and higher viscosities may affect residue patterns. As an exploratory investigation of two under-studied phenotypes, these findings underscore the need for further research to define optimal viscosity targets and support the development of standardized clinical protocols.

## Figures and Tables

**Table 1:** Clinical and demographic profiles of the two patient cohorts.

	Structural OD	Mild OD
No. participants	25	25
Age (years)	81.8 ± 7.1	77.4 ± 7.2 *
Female (%)	72.0	44.0 *
BMI (kg/m^2^)	26.8 ± 5.1	26.6 ± 4.0
MNA-sf	11.5 ± 2.9	10.9 ± 2.0
Well-nourished (12–14) (%)	61.9	36.8
At risk (8–11) (%)	33.3	57.9
Malnourished (0–7) (%)	4.8	5.3
Structural impairments	yes	no
Osteophytes (%)	60.0	-
CP bar (%)	20.0	-
Both (%)	20.0	-

Continuous variables expressed as mean ± standard deviation, * *p* ≤ 0.05. BMI indicates body mass index; CP bar, cricopharyngeal bar; MNA-sf, Mini Nutritional Assessment–Short Form; OD, oropharyngeal dysphagia.

**Table 2:** Videofluoroscopy signs of safety and efficacy impairment across the entire study population.

	Structural OD	Mild OD
Impaired efficacy (%)	100.0	100.0
Impaired labial seal (%)	0.0	4.2
Piecemeal deglutition (%)	68.0	70.8
Oral residue (%)	76.0	83.3
Pharyngeal residue (%)	12.0	12.5
Vallecular residue (%)	72.0	75.0
Impaired safety (%)	48.0	33.0
Aspirations (%)	0.0	0.0
Penetrations (%)	48.0	33.0
Mean max. PAS score	1.5 ± 0.5	1.4 ± 0.5

Continuous variables expressed as mean ± standard deviation. PAS indicates penetration–aspiration scale; OD, oropharyngeal dysphagia.

**Table 3:** The timing of the airway protection mechanisms (laryngeal vestibule closure, LVC) and bolus transfer events (upper esophageal sphincter opening, UESO), mean bolus velocity in the pharynx, and the bolus kinetic energy at each viscosity level assessed.

Population Group	OSR and Kinetics	Viscosity Level (mPa·s)					
		<50	100	200	400	800	1600
Structural OD	LVC (ms)	6.7 ± 115.3	26.6 ± 93.8	12.3 ± 78.0	212.0 ± 94.4	01.9 ± 84.6	18.3 ± 82.3
	UESO (ms)	165.9 ± 79.1	57.5 ± 47.1	77.5 ± 49.9	70.7 ± 54.7	88.3 ± 54.4	94.3 ± 57.0
	Mean velocity (m/s)	0.41 ± 0.23	0.36 ± 0.23	0.36 ± 0.20	0.35 ± 0.17	0.33 ± 0.17	0.31 ± 0.19
	Kinetic energy (mJ)	5.7 ± 6.7	4.8 ± 7.1	4.0 ± 5.3	3.9 ± 4.5	3.5 ± 4.4	3.8 ± 5.2
Mild OD	LVC (ms)	257.4 ± 112.5	244.8 ± 100.9	242.9 ± 121.7	242.6 ± 85.0	232.5 ± 91.7	260.8 ± 105.7
	UESO (ms)	189.5 ± 75.5	179.6 ± 38.1	210.8 ± 65.3	205.0 ± 63.4	211.6 ± 62.3	230.0 ± 65.9
	Mean velocity (m/s)	0.42 ± 0.28	0.36 ± 0.21	0.32 ± 0.16	0.33 ± 0.19	0.31 ± 0.16	0.27 ± 0.13
	Kinetic energy (mJ)	6.8 ± 10.7	4.2 ± 6.6	3.2 ± 3.3	3.5 ± 4.8	2.9 ± 3.1	2.3 ± 2.3

Continuous variables expressed as mean ± standard deviation. OSR indicates oropharyngeal swallowing response; OD, oropharyngeal dysphagia.

## Data Availability

The data presented in this study are available on request from the corresponding author. The data are not publicly available due to ethical restrictions.

## Abbreviations

The following abbreviations are used in this manuscript:

BMI	body mass index
CP	cricopharyngeal
CT	clinical trial
LVC	laryngeal vestibule closure
MNA-sf	Mini Nutritional Assessment–Short Form
OD	oropharyngeal dysphagia
OSR	oropharyngeal swallowing response
PAS	Penetration–aspiration scale
SD	standard deviation
TP	thickening product
TQ	Tsururinko Quickly
UESO	upper esophageal sphincter opening
VFS	videofluoroscopy
V-VST	Volume-Viscosity Swallow Test
